# Depletion of myocardial glucose is observed during endotoxemic but not hemorrhagic shock in a porcine model

**DOI:** 10.1186/cc12843

**Published:** 2013-07-25

**Authors:** Michelle S Chew, Kiran Shekar, Björn A Brand, Carl Norin, Adrian G Barnett

**Affiliations:** 1 Dept of Intensive Care and Perioperative Medicine, Skåne University Hospital Malmö, Lund University, Inga Marie Nilssonsgata, S-20502, Sweden; 2 Critical Care Research Group, the Prince Charles Hospital and the University of Queensland, Rode Road, Chermside, Queensland 4032, Australia; 3 Adrian G Barnett: Institute of Health and Biomedical Innovation, School of Public Health & Social Work, Queensland University of Technology, Musk Road, Kelvin Grove, Queensland 4059, Australia; 4 Department of Anaesthesia and Intensive Care, Hallands Hospital Halmstad and Institute for Clinical Sciences Malmö, Lund University, Lasarettsvägen, S-30185, Sweden

**Keywords:** endotoxemia, sepsis, hemorrhage, shock, myocardium, metabolism, microdialysis

## Abstract

**Introduction:**

Metabolic dysfunction is one of the hallmarks of sepsis yet little is known about local changes in key organs such as the heart. The aim of this study was to compare myocardial metabolic changes by direct measurements of substrates, such as glucose, lactate and pyruvate, using microdialysis (MD) in *in-vivo *porcine endotoxemic and hemorrhagic shock. To assess whether these changes were specific to the heart, we simultaneously investigated substrate levels in skeletal muscle.

**Methods:**

Twenty-six female pigs were randomized to three groups: control (C) *n *= 8, endotoxemic shock (E) *n *= 9 and hemorrhagic shock (H) *n *= 9. Interstitial myocardial pyruvate, lactate and glucose were measured using MD. Skeletal muscle MD was also performed in all three groups.

**Results:**

Marked decreases in myocardial glucose were observed in the E group but not in the H group compared to controls (mean difference (CI) in mmol/L: C versus E -1.5(-2.2 to -0.8), *P *<0.001; H versus E -1.1(-1.8 to -0.4), *P *= 0.004; C versus H -0.4(-1.1 to 0.3), *P *= 0.282). Up to four-fold increases in myocardial pyruvate and three-fold increases in lactate were seen in both shock groups with no differences between the two types of shock. There was no evidence of myocardial anaerobic metabolism, with normal lactate:pyruvate (L:P) ratios seen in all animals regardless of the type of shock.

In skeletal muscle, decreases in glucose concentrations were observed in the E group only (mean difference: C versus E -0.8(-1.4 to -0.3), *P *= 0.007). Although skeletal muscle lactate increased in both shock groups, this was accompanied by increases in pyruvate in the E group only (mean difference: C versus E 121(46 to 195), *P *= 0.003; H versus E 77(7 to 147), *P *= 0.032; C versus H 43(-30 to 43), *P *= 0.229). The L:P ratio was increased in skeletal muscle in response to hemorrhagic, but not endotoxemic, shock.

**Conclusions:**

Endotoxemia, but not hemorrhage, induces a rapid decrease of myocardial glucose levels. Despite the decrease in glucose, myocardial lactate and pyruvate concentrations were elevated and not different than in hemorrhagic shock. In skeletal muscle, substrate patterns during endotoxemic shock mimicked those seen in myocardium. During hemorrhagic shock the skeletal muscle response was characterized by a lack of increase in pyruvate and higher L:P ratios.

Hence, metabolic patterns in the myocardium during endotoxemic shock are different than those seen during hemorrhagic shock. Skeletal muscle and myocardium displayed similar substrate patterns during endotoxemic shock but differed during hemorrhagic shock.

## Introduction

Imbalance in myocardial energy metabolism has been proposed as one of the mechanisms behind cardiac dysfunction associated with sepsis [1,2]. Despite the fact that myocardial utilization of energy substrates and contractility are inextricably linked [3], the extent of metabolic changes in the myocardium and whether they may be a cause of cardiac dysfunction in sepsis remain undefined. The myocardium has a unique ability to utilize multiple substrates for energy production. Free fatty acids (FFAs) are the major fuel for the heart (60% to 100%) with a lesser contribution from glucose and lactate (0 to 20% each) [4]. In patients with septic shock changes in myocardial metabolism have been demonstrated [5] with reported increases in myocardial lactate uptake and decreases in free fatty acid and glucose utilization. However, the substrates involved in myocardial metabolism have generally been measured indirectly using coronary sinus sampling or in *ex-vivo *heart preparations. Further, it is not known if there is a specific pattern of changes for sepsis and if these only apply to cardiac muscle. Impairment of glucose metabolism has been demonstrated in experimental models of septic shock [6,7]. Emerging data suggest that endotoxemic shock may induce distinct changes in the myocardial metabolism that includes an accelerated aerobic glycolytic process resulting in glucose depletion and accumulation of pyruvate [8].These distinctive changes have also been reported in skeletal muscle during endotoxemic shock but not during hemorrhagic shock [8,9]. Possible explanations for this include catecholamine overdrive resulting in increased Na^+ ^K^+ ^ATPase activity, mitochondrial dysfunction and pyruvate dehydrogenase inhibition. It is likely that endotoxemia, hemorrhage and other mechanisms of shock have variable effects on cardiac and skeletal muscle metabolism and this may have implications for clinical management of these conditions. This is highly relevant as approximately 15% of deaths related to septic shock have been attributed to myocardial depression [10].

Carbohydrate metabolism and its regulation in the myocardium is complex and is dependent on arterial substrate levels, coronary blood flow, hormonal levels in the blood, nutritional reserve of the heart and the inotropic state [11]. In the stressed heart, there is a switch from FFAs to other energy substrates as the preferred metabolic fuel [12]. This metabolic flexibility may confer an adaptive advantage to an organ with high energy requirements; indeed, loss of this flexibility leads to maladaptation in conditions such as chronic heart failure and diabetic cardiomyopathy. Little is known about myocardial energetics in sepsis. The absence of demonstrable myocardial ischemia in multiple studies [8,13] during sepsis and shock raises important questions about myocardial energy metabolism during shock states that are often associated with cardiac dysfunction. Our current understanding of myocardial metabolism is largely based on studies that used global markers or measurements from regional blood samples. There are few studies that have used microdialysis (MD) techniques to describe changes in cardiac metabolism. MD provides a unique opportunity to study temporal changes directly in tissues in response to a physiological insult and is a novel way of studying metabolism without the need for repeated biopsies or inference from blood samples. Most of the available myocardial MD studies are limited to conditions of cardiac ischemia, infarction and cardiopulmonary bypass and only a few have been published within the setting of septic or hemorrhagic shock. The understanding of myocardial metabolism in shock states is important as it may lead to pharmacological interventions that can improve cardiac performance by regulating the energy mechanisms of the heart.

The aim of this study was to compare the metabolic changes in the myocardium using MD in porcine models of shock resulting from endotoxemia and hemorrhage. To assess whether these changes were specific to the heart, we simultaneously investigated substrate levels in skeletal muscle.

## Materials and methods

The study was approved by the Swedish National Board for Animal Experimentation (Case number M131-09) and conforms to the guidelines laid out in the Guide for the Care and Use of Laboratory Animals from the National Research Council. Twenty-six female pigs, weighing 32 to 41 kg, were used in the study and were randomized to three groups: control (C) *n *= 8, endotoxemic shock (E) *n *= 9 and hemorrhagic shock (H) *n *= 9.

### Anesthesia, mechanical ventilation and fluids

The porcine endotoxemic model has been previously described [8]. After premedication with xylazine 2 mg/kg IM and ketamine 20 mg/kg IM, general anesthesia was induced with thiopentone 5 mg/kg. General anesthesia was maintained with ketamine 5 mg/kg/hr, midazolam 0.5 mg/kg/hr and fentanyl 10 mcg/kg/hr IV. All animals were intubated and mechanically ventilated with a Siemens 900 Ventilator (Siemens Elema, Stockholm, Sweden). Ventilator settings were adjusted to maintain PaCO2 between 4.5 and 6.0 kPa and SaO2 over 95%. Ringer's acetate 10 ml/kg/hr was infused to compensate for fluid loses and an additional 500 ml was given before the induction of shock.

### Monitoring

All animals were monitored with (three-lead ECGs,) invasive arterial blood pressure measurement, SpO2 and hourly urine output. Arterial blood gases were drawn every two hours for adjustment of ventilator settings.

### Instrumentation

The external jugular veins were exposed bilaterally by surgical cut-down. A central venous catheter was inserted for drug and fluid infusion. The contralateral external jugular vein was used for the insertion of a pulmonary artery catheter (PAC). This was used for hourly measurements of central venous pressure (CVP), mean pulmonary arterial pressure (MPAP) and pulmonary capillary wedge pressure (PCWP).

The left or right carotid artery was also exposed by surgical cut-down, and an arterial catheter was inserted for blood gas sampling.

The left or right femoral artery was also exposed surgically and an arterial catheter was inserted for bleed-out in the animals assigned to the hemorrhagic shock groups. A midline thoracotomy was performed and the pericardium divided, suspending the heart in a pericardial sling. A MD catheter (CMA 20, CMA Microdialysis, Solna Sweden) was inserted into the anterior surface of the myocardium, immediately adjacent and parallel to the diagonal branches of the left anterior descending artery. The catheters were perfused with Ringer's acetate at 0.3 uL/minute. MD catheters were left *in-situ *for at least one hour for stabilization before microdialysate samples were collected.

The pericardial cavity was filled with saline solution and ultrasound gel to provide acoustic coupling for transepicardial echocardiographic imaging.

Via direct surgical exposure a MD catheter (CMA 20, CMA Microdialysis, Solna, Sweden) was inserted into the gluteus maximus muscle. The catheters were perfused with Ringer's acetate at 0.3 uL/minute. Microdialysis catheters were left *in-situ *for at least one hour for stabilization before microdialysate samples were collected.

The urinary bladder was catheterized using a suprapubic approach and urine output (UO) measured hourly.

### Induction of endotoxemia

Endotoxemia was induced using *Escherichia coli *lipopolysaccharide (LPS O111:B4, product code L2630, Sigma Aldrich, St Louis, MO, USA) at an infusion rate of 5 ug/kg/hour for the first hour followed by 2.5 ug/kg/hour for the remaining duration of the experiment.

### Induction of hemorrhagic shock

A bleed out of 30% blood volume was made via a femoral arterial catheter assuming a blood volume of 76 ml/kg for all pigs [14]. Bleed out was performed at a rate of 2 ml/kg/minute for five minutes, followed by 1 ml/kg/minute for 12.5 minutes.

### Measurements

The following systemic hemodynamic measurements were recorded hourly: mean arterial pressure (MAP), MPAP, PCWP, CVP, mixed venous oxygen saturation (SmvO2), cardiac index (CI) and systemic vascular resistance index (SVRI). The following parameters were calculated: stroke volume index (SVi), oxygen delivery index (DO2i), oxygen consumption index (VO2i), left ventricular stroke work index (LVSWI), right ventricular stroke work index (RVSWI) and oxygen extraction ratio (O2ER).

Urine output was recorded hourly.

Microdialysate samples in the myocardial and skeletal muscle interstitium were collected every hour for the measurement of interstitial glucose, lactate and pyruvate and for the calculation of lactate to pyruvate ratios (L:P). Analyses of these metabolites were made on an ISCUS^flex ^microdialysis analyzer {AU Query: Please indicate the manufacturer of the microdialysis analyzer - city, state (if applicable), country.}(M-dialysis, Solna, Sweden) according to the manufacturer's instructions. This analyzer uses enzymatic reagents and colorimetric assays, with an assay imprecision of <4%.

Transthoracic echocardiographic images of the heart were obtained from the apex using a direct epicardial approach. Four-chamber and two -chamber views are obtained. Left ventricular (LV) systolic function was evaluated using ejection fraction by eyeballing (EF), velocity time integral at the left ventricular outflow tract (VTI) and the average of septal and lateral tissue Doppler velocities (TDI). LV diastolic function was evaluated using the mitral early to late inflow velocity ratio (E/A) and mitral early inflow to tissue Doppler velocity ratio (E/é). Right ventricular function was assessed as the tricuspid annular plane systolic excursion (TAPSE) and the pressure gradient between the right atrium and ventricle (TR).

### Statistics

A mixed model analysis with a random intercept for each pig was used to control for between-pig differences. We modeled each response over time with group (C, E or H) as independent variables. Baseline response was included as a covariate in order to control for regression to the mean. No linear assumptions were made for the change over time. We calculated the average difference between groups over all times (with a 95% confidence interval) and the maximum difference over hours one to six. *P*-values less than 0.05 were considered statistically significant. Skewed data were log-transformed. All analyses were conducted using R (http://www.r-project.org) and Sigma Stat version 3.5 (Systat Software, San Jose, California, USA).

## Results

### Hemodynamic parameters

Hemodynamic alterations in the E group were characterized by significant increases in heart rate (HR) and MPAP and decreases in MAP, SVI and LVSWI compared to the control group. RVSWI increased, reflecting increases in pulmonary arterial pressures, a finding supported by echocardiography parameters. There were trends to decreased SmvO2 but these changes were short-lived, reaching statistical significance only within the first two hours of endotoxemia. Arterial lactate concentrations increased significantly, up to three-fold compared to controls. Despite the decrease in MAP and increases in HR and arterial lactate concentration (A-lac), DO2i, VO2i and O2ER were relatively preserved. In the hemorrhage group significant increases were observed in O2ER and decreases in MAP, CVP, PCWP, DO2i, LVSWI and RVSWI compared to C and E animals. A-lac increased 2.5-fold.

There were no significant changes in arterial glucose concentrations in any of the groups over time nor between the groups (data not shown).

### Echocardiography

There were significant decreases in LV systolic function as assessed by EF in the E group compared to the C and H groups. EF increased slightly in the H group and decreased in the E group, with statistically significant differences between the two shock groups from four hours post endotoxemia (t4) onwards. LV diastolic function, measured as E/A and E/é remained unchanged in all three groups.

VTI significantly decreased in the H group and was significantly different from the C group. VTI was also decreased in the E group, but this did not reach statistical significance (*P *= 0.056).

RV function measured by TAPSE was decreased in the E and but not the C or H groups with maximal differences towards the end of the observation period. Reflecting decreased RV function, TR was higher in the E group, differing significantly from the H group.

Hemodynamic and echocardiographic parameters are shown in Table 1.

**Table 1:** Hemodynamic and echocardiographic parameters

		Mean difference (CI)	*P*(mean)	Max difference (CI)	*P*(max)
HR (bpm)	C vs H	12.7(-1.7 to 27.2)	NS(0.081)	18.4(-1.3 to 38.2)	NS(0.066)
	C vs E	16.3(2.0 to 30.5)	0.027	25.6(6.0 to 45.2)	0.013
	H vs E	3.5(-10.6 to 17.7)	NS(0.609)	N/A	NS(0.151)
MAP (mmHg)	C vs H	-34.0(-44.7 to -23.3)	<0.001	-48.6(-61.4 to -35.8)	<0.001
	C vs E	-9.8(-20.6 to 1.1)	NS(0.075)	-29.9(-42.8 to -17.0)	<0.001
	H vs E	24.3(14.2 to 34.3)	<0.001	29.1(16.6 to 41.6)	<0.001
MPAP (mmHg)	C vs H	-3.5(-7.0 to -0.1)	0.047	unable to identify sig. changes	NS(0.107)
	C vs E	10.9(7.2 to 14.7)	<0.001	19.4(14.4 to 24.4)	<0.001
	H vs E	14.5(10.8 to 18.1)	<0.001	23.3(18.4 to 28.1)	<0.001
CVP (mmHg)	C vs H	-1.8(-3.4 to -0.2)	0.029	-2.4(-4.3 to -0.4)	0.020
	C vs E	-0.3(-1.8 to 1.3)	NS(0.722)	N/A	NS(0.295)
	H vs E	1.5(0.1 to 3.0)	0.041	2.9(1.0 to 4.7)	0.004
PCWP (mmHg)	C vs H	-2.4(-4.0 to -0.9)	0.004	-3.0(-5.0 to -0.9)	0.008
	C vs E	0.5(-1.0 to 2.1)	NS(0.464)	2.9(0.9 to 5.0)	0.007
	H vs E	3.0(1.5 to 4.4)	<0.001	5.1(3.2 to 7.1)	<0.001
SVI (ml/m2)	C vs H	-8.2(-12.5 to -3.8)	0.001	-10.5(-16.6 to -4.5)	0.002
	C vs E	-6.0(-10.3 to -1.7)	0.008	-7.8(-13.8 to -1.7)	0.014
	H vs E	2.1(-2.3 to 6.5)	NS(0.322)	5.4(-0.6 to 11.4)	NS(0.076)
SmvO2 (%)	C vs H	-23.8(-32.9 to -14.7)	<0.001	-33.4(-45.4 to -21.4)	<0.001
	C vs E	-6.1(-15.2 to 3.0)	NS(0.178)	-14.9(-26.9 to -2.9)	0.017
	H vs E	17.7(9.2 to 26.2)	<0.001	19.2(8.0 to 30.4)	0.002
A-Lac (mmol/L)	C vs H	2.3(0.6 to 4.1)	0.011	2.7(0.7 to 4.7)	0.010
	C vs E	2.8(1.0 to 4.6)	0.003	3.3(1.3 to 5.3)	0.003
	H vs E	0.5(-1.1 to 2.1)	NS(0.529)	N/A	NS(0.188)
DO2i (ml/min/m2)	C vs H	-96.2(-157.2 to -35.2)	0.004	-113.6(-185.7 to -41.5)	0.004
	C vs E	1.6(-61.5 to 64.6)	NS(0.959)	N/A	NS(0.536)
	H vs E	97.8(35.5 to 160.0)	0.004	120.1(47.0 to 193.1)	0.003
VO2i (ml/min/m2)	C vs H	0.7(-28.8 to 30.2)	NS(0.962)	N/A	NS(0.275)
	C vs E	14.8(-17.0 to 46.6)	NS(0.343)	N/A	NS(0.242)
	H vs E	14.1(-16.6 to 44.8)	NS(0.349)	N/A	NS(0.210)
O2ER	C vs H	0.2 (0.2 to 0.3)	<0.001	0.3(0.2 to 0.4)	<0.001
	C vs E	0.1(0.0 to 0.2)	NS(0.119)	0.1(0.0 to 0.2)	0.029
	H vs E	-0.2(-0.3 to -0.1)	<0.001	-0.2(-0.1 to -0.3)	0.001
LVSWI (g.mm2)	C vs H	-16.9(-22.6 to -11.2)	<0.001	-24.4(-31.4 to -17.3)	<0.001
	C vs E	-9.1(-14.7 to -3.6)	0.003	-17.2(-24.2 to -10.3)	<0.001
	H vs E	7.8(2.3 to 13.3)	0.008	9.5(2.3 to 16.7)	0.012
RVSWI (g.mm2)	C vs H	-2.2(-4.0 to -0.5)	0.016	-2.6(-4.9 to -0.3)	0.031
	C vs E	2.0(0.2 to 3.8)	0.027	4.9(2.6 to 7.2)	<0.001
	H vs E	4.2(2.5 to 6.0)	<0.001	7.5(5.2 to 9.7)	<0.001
EF (%)	C vs H	2.9(-1.8 to 7.5)	NS(0.210)	N/A	NS(0.263)
	C vs E	-9.5(-14.4 to -4.7)	0.001	-10.5(-16.6 to -4.5)	0.002
	H vs E	-12.4(-17.0 to -7.8)	<0.001	-13.1(-18.9 to -7.4)	<0.001
TDI (mm)	C vs H	-0.5(-1.1 to 0.2)	NS(0.143)	-1.0(-2.1 to 0.0)	NS(0.055)
	C vs E	-0.4(-1.1 to 0.2)	NS(0.182)	-1.0(-2.2 to 0.2)	NS(0.092)
	H vs E	0.0(-0.6 to 0.7)	NS(0.943)	N/A	NS(0.462)
VTI (cm)	C vs H	-4.0(-6.3 to -1.8)	0.001	-5.7(-8.4 to -3.1)	<0.001
	C vs E	-2.2(-4.5 to 0.1)	NS(0.056)	-2.8(-5.6 to 0.0)	0.048
	H vs E	1.8(-0.4 to 4.0)	NS(0.099)	3.8(1.3 to 6.3)	0.005
TAPSE (mm)	C vs H	-1.2(-3.5 to 1.0)	0.268	-2.7(-5.5 to 0.1)	NS(0.060)
	C vs E	-3.0(-5.1 to -0.9)	0.008	-4.0(-6.7 to -1.3)	0.006
	H vs E	-1.8(-3.9 to 0.4)	NS(0.105)	-3.3(-5.9 to -0.6)	0.018
TR (mmHg)	C vs H	-1.7(-10.7 to 7.3)	NS(0.695)	N/A	NS(0.441)
	C vs E	8.3(-0.8 to 17.4)	NS(0.070)	10.3(-0.4 to 21.1)	NS(0.059)
	H vs E	10.0(2.0 to 18.1)	0.018	14.5(5.0 to 24.0)	0.005
E/A	C vs H	0.1(-0.2 to 0.4)	NS(0.491)	N/A	NS(0.098)
	C vs E	-0.3(-0.7 to 0.0)	0.034	-0.5(-0.8 to -0.1)	0.015
	H vs E	-0.5(-0.8 to -0.2)	0.005	-0.6(-1.0 to -0.2)	0.004
E/é	C vs H	2.5(0 to 5.1)	0.051	4.0(0.3 to 7.7)	0.036
	C vs E	-1.5(-4.1 to 1.1)	NS(0.236)	N/A	NS(0.066)
	H vs E	-4.0(-1.6 to -6.5)	0.002	-5.5(-8.9 to -2.1)	0.003

Mean and maximum differences from baseline and CI are given. A positive mean or maximum difference indicates increased values in H or E groups compared to controls (C versus H and C versus E) and vice versa. Similarly, for H versus E a positive mean or maximum difference indicates an increase in the E group compared to the H group. *P*(mean) refers to intergroup differences over time using mixed models analysis. *P*(max) refers to *post-hoc *analyses at the time of maximal difference and are given as unadjusted *P*-values. A-lac, arterial lactate concentration; C, control group; CVP, central venous pressure; DO2i, oxygen delivery index; E; endotoxemic group; E/A, mitral early to late flow ratio; E/é, mitral early flow to tissue Doppler velocity ratio; EF, ejection fraction; H, hemorrhagic group; HR,heart rate; LVSWI, left ventricular stroke work index; MAP, mean arterial pressure; MPAP, mean pulmonary arterial pressure; NS, non-significant; O2ER, oxygen extraction ratio; PCWP, pulmonary capillary wedge pressure; RVSWI, right ventricular stroke work index; SmvO2, mixed venous oxygen saturation; SVI, stroke volume index; TAPSE, tricuspid annular plane systolic excursion; TDI, average (septal and lateral) tissue Doppler velocity; TR, right atrial-right ventricular pressure gradient measured using tricuspid regurgitation; VO2i, oxygen consumption index; VTI, velocity time integral at the left ventricular outflow tract.

### Microdialysis

#### Myocardial metabolites in controls, endotoxemic and hemorrhagic shock

Mean and maximum differences between the groups are shown in Table 2.

**Table 2:** Microdialysis parameters for myocardium and skeletal muscle

	Myocardium				Skeletal muscle			
	**Mean difference(CI)**	***P*(mean)**	**Max difference(CI)**	***P*(max)**	**Mean difference (CI)**	***P*(mean)**	**Max difference (CI)**	***P*(max)**

Glucose					Glucose			
C vs H	-0.4(-1.1 to 0.3)	0.282	-1.2(-2.6 to 0.3)	0.109	-0.4(-1.0 to 0.2)	0.155	-0.8(-1.5 to 0)	0.060
C vs E	-1.5(-2.2 to -0.8)	<0.001	-2.5(-4.0 to -0.9)	0.003	-0.8(-1.4 to -0.3)	0.007	-1.4(-2.2 to -0.6)	0.002
H vs E	-1.1(-1.8 to -0.4)	0.004	-1.4(-2.8 to 0.0)	0.043	-0.4(-1.0 to 0.1)	0.111	-1.0(-1.7 to -0.2)	0.016
Lactate					Lactate			
C vs H	2.5(0.5 to 4.4)	0.016	3.4(1.1 to 5.7)	0.006	2.0(0.1 to 3.9)	0.041	2.8(0.5 to 5.0)	0.018
C vs E	2.2(0.2 to 4.1)	0.032	2.7(0.4 to 4.9)	0.013	1.7(-0.2 to 3.7)	0.083	2.9(0.5 to 5.3)	0.020
H vs E	-0.3(-2.2 to 1.6)	0.740	-1.7(-3.8 to 0.5)	0.118	-0.3(-2.2 to 1.6)	0.743	1.3(-1.0 to 3.6)	0.243
Pyruvate					Pyruvate			
C vs H	120(65 to 176)	<0.001	148(65 to 232)	0.001	43(-30 to 43)	0.229	60(-37 to 157)	0.212
C vs E	136(80 to 192)	<0.001	186(104 to 268)	<0.001	121(46 to 195)	0.003	238(132 to 345)	<0.001
H vs E	15(-34 to 65)	0.525	54(-28 to 136)	0.184	77(7 to 147)	0.032	210(109 to 311)	<0.001
L:P					L:P			
C vs H	6.0(-1.7 to 13.7)	0.122	9.2(0.6 to 17.8)	0.087	0.4(0.2 to 1.0)	0.193	0.6(-0.1 to 1.3)	0.092
C vs E	3.7(-4.6 to 12.0)	0.363	6.7(-2.5 to 15.9)	0.142	-0.2(-0.8 to 0.5)	0.577	-0.6(-1.3 to 0.2)	0.122
H vs E	-2.3(-9.8 to 5.3)	0.540	-6.0(-14.3 to 2.2)	0.144	-0.6(-1.2 to 0)	0.061	-0.9(-1.60.2)	0.014

Mean and maximum differences from baseline and CIs are given. A positive mean or maximum difference indicates increased values in H or E groups compared to controls (C versus H and C versus E) and vice versa. Similarly, for H versus E a positive mean or maximum difference indicates an increase in the E group compared to the H group. *P*(mean) refers to intergroup differences over time using mixed models analysis. *P*(max) refers to *post-hoc *analyses at the time of maximal difference and are given as unadjusted *P*-values.

**Figure 1:**
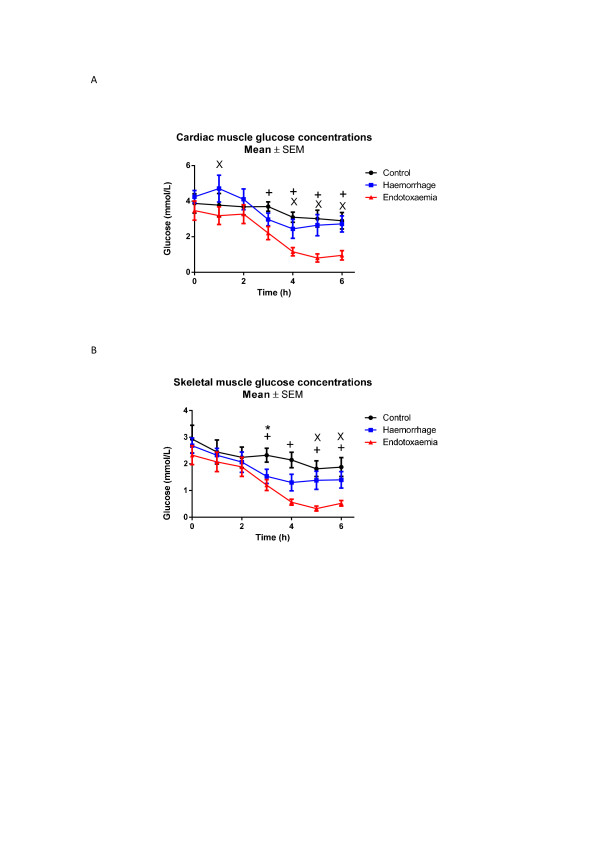
****A**) Myocardial and **B**) skeletal muscle glucose concentrations**. t0 refers to baseline measurements prior to induction of endotoxemic or hemorrhagic shock. Significant decreases over time were seen in the myocardium during endotoxemic but not hemorrhagic shock compared to controls (*P *<0.001). There was a similar decrease in skeletal muscle during endotoxemic shock (*P *= 0.007) but not during hemorrhagic shock. Significant differences for individual time points are marked * for C versus H, ^+ ^for C versus E, ^x ^for H versus E. C, control group; E, endotoxemic group; H, hemorrhagic group.

**Figure 2:**
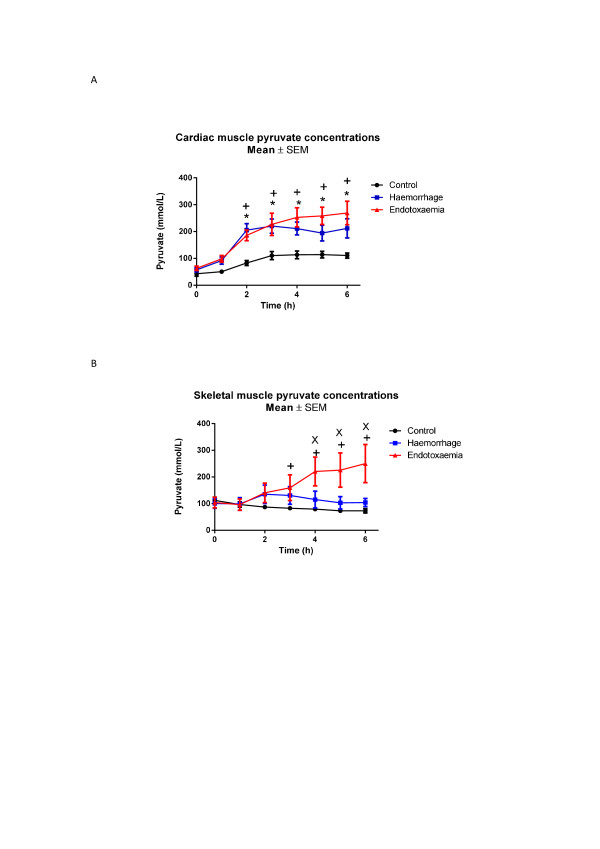
****A**) Myocardial and **B**) skeletal muscle pyruvate concentrations**. t0 refers to baseline measurements prior to induction of endotoxemic or hemorrhagic shock. Significant increases over time were seen in myocardium in response to both endotoxemic and hemorrhagic shock compared to controls. In skeletal muscle increases in pyruvate over time were seen in endotoxemic shock (*P *= 0.003 compared to C group, *P *= 0.032 compared to H group), but no changes were seen during hemorrhagic shock. Significant differences for individual time points are marked * for C versus H, ^+ ^for C versus E, ^x ^for H versus E. C, control group; E, endotoxemic group; H, hemorhagic group.

**Figure 3:**
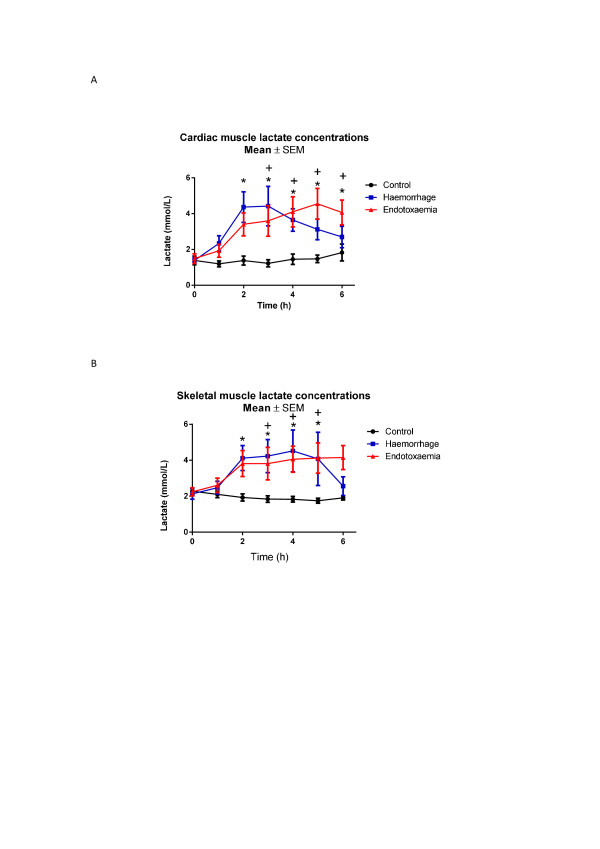
****A**) Myocardial and **B**) skeletal muscle lactate concentrations**. t0 refers to baseline measurements prior to induction of endotoxemic or hemorrhagic shock. Significant increases over time in cardiac and skeletal muscle lactate were seen in both endotoxemic and hemorrhagic shock. Significant differences for individual time points are marked * for C versus H, ^+ ^for C versus E, ^x ^for H versus E. C, control group; E, endotoxemic group; H, hemorrhagic group.

**Figure 4:**
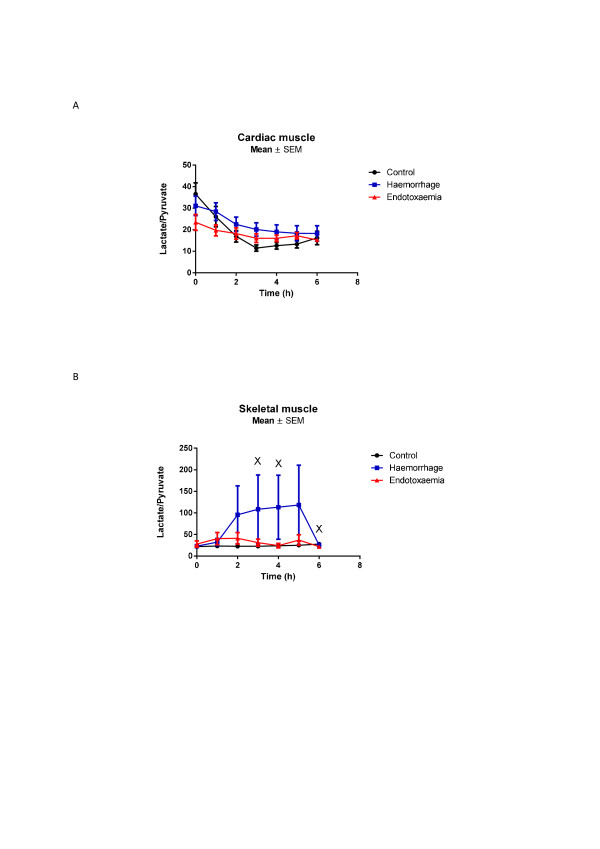
****A**) Myocardial and **B**) skeletal muscle L:P ratios**. t0 refers to baseline measurements prior to induction of endotoxemic or hemorrhagic shock. No differences over time were observed during endotoxemic shock. In contrast, hemorrhagic shock tended to increase L:P in skeletal muscle (*P *= 0.061 for mean difference, *P *= 0.014 for maximum difference compared to E group). Significant differences for individual time points are marked * for C versus H, ^+ ^for C versus E, ^x ^for H versus E. C, control group; E, endotoxemic group; H, hemorrhagic group; L:P, lactate pyruvate ratio.

Marked decreases in myocardial glucose were observed in the E group towards the end of the study period (nadir 0.6 (0.5 to 0.8) mmol/L, *P *<0.001 compared to baseline) whereas they remained relatively stable in the H group (Figure 1a). There were significant mean differences between C versus E (*P *<0.001) and H versus E groups (*P *= 0.004). Post-hoc analysis revealed significant differences between C and E animals and between H and E animals from t3 onwards. No difference was seen for the C versus H group. Increases in myocardial pyruvate were seen in all animals, with four-fold increases observed in H and E shock groups (Figure 2a). There were significant mean differences between the C and E groups (*P *<0.001) and between the C and H groups (*P *<0.001) but not between the H and E animals. Myocardial lactate levels also increased in both shock groups (Figure 3a). Significant mean differences were seen between the C and E groups (*P *= 0.032) and between the C and H groups (*P *= 0.016). There were no differences between the E and H groups. The myocardial-A-lac gradient was significantly increased in the E compared to the H group (mean difference H versus E 0.7 (CI 0 to 1.3) mmol/L, *P *= 0.04) but was not significantly different from the C group (mean difference C versus E -0.3 (CI -1.0 to 0.3) mmol/L, *P *= 0.299). The L:P ratio over time decreased significantly in all animals, indicating no evidence of myocardial anaerobic metabolism (Figure 4a).

#### Comparison between skeletal and cardiac muscle

During endotoxemic shock, myocardial and skeletal muscle glucose were both significantly decreased compared to controls with minimum concentrations reached at t5 (myocardium: nadir 0.6 (0.5 to 0.8) mmol/L, skeletal muscle: nadir 0.4 (0.3 to 1.0) mmol/L; P <0.001 compared to baseline for both organs). In contrast, hemorrhagic shock did not significantly decrease myocardial or skeletal muscle glucose compared to controls (Table 2, Figure 1a and 1b). Lactate increased significantly in both skeletal and cardiac muscles, for both E and H groups with peak levels reached at different times (E group: myocardium 3.5 versus skeletal muscle 3.9 mmol/L at t = 6; H group: myocardium 3.5 versus skeletal muscle 3.0 mmol/L at t = 3) (Figure 3a and 3b). Whereas myocardial pyruvate levels increased in both types of shock, skeletal muscle pyruvate increased only in response to endotoxemic shock (mean difference C versus E *P *= 0.003, C versus H *P *= 0.229, H versus E *P *= 0.032) (Table 2, Figure 2b). L:P ratios decreased in cardiac muscle in all animals (Figure 4a). In contrast L:P tended to increase over time for the hemorrhagic shock animals in skeletal muscle. For the H group compared to the E group there was a mean difference in L:P ratio of 0.6 (0 to 1.2), *P *= 0.061 and a maximum difference -0.9 (-1.6 to -0.2), *P *= 0.014 at t4 (Figure 4b).

## Discussion

To our knowledge this is the first reported study comparing myocardial metabolism using MD in models of hemorrhagic and endotoxemic shock. The most striking finding of this study is the precipitous decrease in myocardial glucose concentrations to near-zero levels in endotoxemic, but not hemorrhagic, shock. Despite the decrease in myocardial glucose concentrations during endotoxemia, similar changes in myocardial lactate and pyruvate concentrations were measured in both hemorrhagic and endotoxemic shock. Therefore, endotoxemic and hemorrhagic shock seem to be characterized by a different myocardial glucose response but similar pyruvate and lactate responses.

Next, we asked if these changes were specific to the myocardium. In skeletal muscle, endotoxemia also induced decreases in glucose, increases in lactate, pyruvate and preserved L:P ratios. In contrast hemorrhagic shock did not increase skeletal muscle pyruvate. The resultant L:P ratio in skeletal muscle was increased indicative of anaerobic metabolism. These results demonstrate that the skeletal muscle responds differently to the different types of shock. Taken together, they suggest that the heart and skeletal muscle have different metabolic signatures that are dependent on the type of shock.

While FFAs are known to be the preferred energy substrate for the heart [12], under stressed conditions other energy substrates may be preferentially used during oxidative metabolism. Sustained, enhanced non-oxidative glucose utilization has also been reported in models of ischemia-reperfusion [15]. There is indication that an enhanced utilization of exogenous glucose improves cardiac function during hypoxic states without increasing total glycolytic flux or tissue high-energy phosphate levels, representing a novel cardioprotective mechanism [16]. This 'metabolic flexibility' has been thought to confer an advantage to the heart where there is a constant and high energy requirement. Indeed, loss of metabolic flexibility has been shown to be deleterious in chronic conditions, such as hypertension induced congestive heart failure and diabetic cardiomyopathy, in experimental models [11,17].

Another relevant question identified by these findings is whether the decrease in myocardial glucose concentrations may be associated with cardiac dysfunction. While we demonstrated significant impairment in both left and right ventricular function measured using echocardiography, all of the measured indices are, to different extents, preload dependent. Hence, we refrained from any further analysis of the relationship between cardiac function and metabolic parameters. A longer observation period with low concentrations of myocardial glucose may have revealed a depletion of myocardial energy stores and been associated with further decreases in cardiac function. There was a concurrent rise in myocardial pyruvate, suggesting either the conversion of glucose or lactate into pyruvate for use in the tricarboxylic acid cycle.

This study supports the concept of accelerated aerobic glycolysis in the myocardium where the rate of glucose metabolism exceeds the oxidative capacity of the mitochondria. Thus, glucose is converted to pyruvate, whose concentration rises and drives the production of lactate with resultant normal L:P ratios. This was particularly observed during endotoxemic shock with about four-fold increases in pyruvate, accompanying increases in interstitial lactate and low L:P ratios. This increase in pyruvate may be an adaptive mechanism since pyruvate in supraphysiological concentrations has been shown to increase cardiac contractile performance and myocardial energy state and protect the myocardium from ischemia-reperfusion injury and oxidant stress [18]. A key link here may be pyruvate dehydrogenase (PDH) kinase. PDH kinase inhibits PDH and, hence, pyruvate decarboxylation, a key and irreversible step in carbohydrate oxidation. PDH kinase activity is increased by low carbohydrate reserves [4,11,19], consistent with the findings of high interstitial pyruvate and low interstitial glucose in this study.

There was no evidence of myocardial ischemia during shock from endotoxemia or hemorrhage despite significant hemodynamic alterations particularly in the latter group. Hence, further support is provided for Levy *et al*.'s landmark studies [9,20,21] which demonstrated the concept of accelerated glycolysis via Na+K+ATPase stimulation in skeletal muscle. We now show that the same process occurs in the heart, an organ with high metabolic demands and accounting for only a small amount of total body lactate production.

Myocardial metabolism of energy substrates is altered in the setting of increased cardiac work and inflammation [5-7,17]. In isolated perfused rat hearts, impaired glucose metabolism was demonstrated when alternative energy substrates were present, specifically glycolysis and glycogen synthesis were impaired [7]. In patients with septic shock, Dhainaut *et al*. demonstrated increases in myocardial lactate uptake and decreases in free fatty acid and glucose utilization measured in coronary sinus blood [5]. The increase in lactate uptake is notable, since the study could not identify whether this shift to lactate extraction was merely a reflection of increased A-lac levels. A positive lactate gradient in the endotoxemia group compared to the hemorrhage group suggests that the myocardium itself produces lactate during endotoxemia and may be part of a metabolic switch in order to ensure substrate availability to this organ. However, we also found that increased interstitial lactate concentrations reflected arterial levels in both types of shock. The finding of increased lactate and pyruvate is consistent with Taegtmeyer's concept of the heart being a 'metabolic omnivore'[11], switching energy substrates to maintain metabolic flexibility. This protective mechanism allowing the body to use other substrates for the generation of energy in the face of increased metabolic needs could explain why cardiac function was preserved in our study despite hemodynamic compromise. Indeed enzymatic depletion of lactate has been shown to cause decreased myocardial performance, cardiovascular collapse and increased mortality in a rat model of endotoxic shock [20]. An attractive hypothesis is that of a switch from fatty acid to glucose metabolism as a mechanism for cardioprotection, as seen in hibernating hearts [11].

There are a number of limitations to this study. Firstly, there are important differences between endotoxemic and septic shock and findings here may not be generalizable to patients with sepsis. However, this was a proof-of-concept study and endotoxemia is a valid model for demonstrating that myocardial concentrations of major energy substrates are affected by major inflammatory insults. The endotoxemic and hemorrhagic shock groups had different temporal hemodynamic profiles with endotoxic shock animals experiencing less severe decreases in MAP and SmvO_2, _and greater increases in MPAP and A-lac levels. Hence, the two groups are difficult to compare and the findings here should be interpreted cautiously. The increases in myocardial pyruvate levels in the control group were unexpected and we speculated if it could have been a result of anesthesia, sternotomy and fluid resuscitation with Ringer's lactate. However, all animals were subjected to the same anesthesia and surgical procedures. Further increases in pyruvate were only seen in cardiac muscle. In a previous (unpublished) LPS dose-finding study we tested resuscitation with 0.9% saline (*n *= 4) and 5% albumin (*n *= 3), with similar results seen for pyruvate measurements in the myocardium. Hence, it is unlikely that this phenomenon is due to the administration of Ringer's acetate. It would be relevant to study the contribution of other energy substrates in the heart, such as FFAs, ketones and glycogen, which were not available using the present microdialysis technique. In line with this, it would also have been relevant to measure myocardial ATP levels. Further, while we have been able to measure substrate availability using microdialysis, we did not study their uptake or oxidation *per se*. Hence, any changes in substrate concentrations observed here cannot be ascribed to changes in delivery, uptake or consumption. This would be relevant in future research and requires measurement of regional flow and the use of labeled isotope which was beyond the scope of the present study. Finally, the observation time for this study was six hours post-induction of shock, and the changes here represent only the acute situation. These findings may be altered in the setting of hypodynamic, endotoxemic shock. A longer observation period may have revealed different results and allowed the investigation of prolonged interstitial metabolic changes on cardiac function. In addition, there may have been differences between the right and left heart that were beyond the scope of this study.

Although we have used currently accepted echocardiographic indices for the measurement of systolic and diastolic function, all echo data provided in the present study are preload, afterload and HR dependent. It is, therefore, difficult, if at all possible, to differentiate between direct myocardial systolic and diastolic effects of endotoxemia from secondary effects caused by endotoxemia-induced changes in preload, afterload or heart rate.

Notwithstanding the limitations above, microdialysis provides a unique possibility for studying the temporal changes in metabolism directly in tissues. We demonstrate these changes for the first time in the heart, comparing it to a different metabolically active organ, and in two different types of shock. A logical extension to this study would be to investigate changes during hypodynamic septic shock and during overt myocardial dysfunction, to monitor responses during resuscitation, as well as to extend MD studies to other organs.

## Conclusions

Endotoxemic and hemorrhagic shock induce profound changes in myocardial energy substrates, characterized by up to four-fold increases in interstitial pyruvate and three-fold increases in interstitial lactate. Endotoxemic, but not hemorrhagic, shock was characterized by a precipitous decrease in myocardial glucose. In skeletal muscle similar patterns were seen during endotoxemic shock with decreases in glucose and increases in pyruvate and lactate concentrations. However, during hemorrhagic shock, the skeletal muscle response differed and was characterized by a lack of increase in pyruvate. Taken together, these findings suggest that the heart and skeletal muscle respond in metabolically distinct fashions in different types of shock.

## Key messages

• Endotoxemic shock causes a severe and precipitous decrease in myocardial glucose concentrations

• Despite the decrease in glucose, myocardial pyruvate and lactate concentrations are simultaneously increased in both types of shock

• Skeletal muscle responds differently with no increases in pyruvate seen in hemorrhagic shock

• Taken together, these findings suggest that the heart responds in a metabolically distinct fashion in endotoxemic and hemorrhagic shock

• The finding of increased lactate and pyruvate is consistent with Taegtmeyer's concept of the heart being a 'metabolic omnivore', switching energy substrates to maintain metabolic flexibility

## Abbreviations

A-lac: arterial lactate; CI: cardiac index; CVP: central venous pressure; DO2i: oxygen delivery index; E/A: mitral early inflow to late (atrial) inflow velocity ratio; E/é: mitral early inflow to tissue doppler velocity ratio. EF: ejection fraction; FFAs: free fatty acids; HR: heart rate; L:P: lactate to pyruvate ratio; LPS: lipopolysaccharide; LVSWI: left ventricular stroke work index; MAP: mean arterial pressure; MD: microdialysis; MPAP: mean pulmonary arterial pressure; O2ER: oxygen extraction ratio; PAC: pulmonary arterial catheter; PCWP: pulmonary capillary wedge pressure; PDH: pyruvate dehydrogenase; RVSWI: right ventricular stroke work index; SmvO2: mixed venous oxygen saturation; SVI: stroke volume index; SVRI: systemic vascular resistance index; TAPSE: tricuspid annular plane systolic excursion; TDI: tissue Doppler velocity; TR: pressure gradient between right atrium and ventricle measured using Doppler; VO2i: oxygen consumption index; VTI: velocity time integral at the left ventricular outflow tract.

## Competing interests

The authors declare that they have no competing interests.

## Authors' contributions

MSC designed the study, collected data, assisted in statistical analysis and drafted the manuscript. BAB was involved in study design, collected data and assisted in statistical analysis. KS assisted in statistical analysis, interpretation of the data and drafted the manuscript. CN was involved in study design, collected data and performed all MD measurements. AGB conducted all statistical analyses, interpreted the data and was involved in drafting the manuscript. All authors have read and approved the final manuscript.
